# ASO Author Reflections: Stratifying Patients for Adjuvant Chemotherapy in Intrahepatic Cholangiocarcinoma: A Novel Approach Using Tumor Burden and Lymph Node Status

**DOI:** 10.1245/s10434-025-17055-9

**Published:** 2025-02-22

**Authors:** Jun Kawashima, Timothy M. Pawlik

**Affiliations:** 1 Department of Surgery, The Ohio State University Wexner Medical Center and James Comprehensive Cancer Center, The Urban Meyer III and Shelley Meyer Chair for Cancer Research, Columbus, OH USA; 2 Department of Gastroenterological Surgery, Yokohama City University School of Medicine, Yokohama, Japan

## Past

Among patients with intrahepatic cholangiocarcinoma (ICC), the role of adjuvant chemotherapy (AC) following curative-intent resection remains a subject of ongoing debate.^1^ While AC has been suggested to reduce tumor recurrence and improve overall survival (OS), its universal application is controversial.^1^ Most of the existing evidence on the efficacy of AC in ICC has been derived from clinical trials that included heterogeneous cohorts encompassing various biliary tract cancers.^2,3^ The results of these trials have been inconsistent, likely owing to multiple contributing factors.^1–3^ One possible explanation is that AC does not exert a uniform effect across all patients with ICC; rather, specific subsets of patients may derive greater benefit from AC than others. Identifying these subsets of patients may enhance clinical decision-making and facilitate a more personalized approach to postoperative therapy.^1^ Therefore, our study aimed to determine which subset of patients with ICC benefited most from AC.^4^

## Present

In the current study, both a higher tumor burden score (TBS) [hazard ratio (HR) 0.95, 95% CI 0.91–1.00, and *p* = 0.033) and metastatic lymph node status (HR 0.58, 95% CI 0.38–0.89, and *p* = 0.014) demonstrated strong interactions with the survival benefit associated with AC. These findings suggested that patients with a greater tumor burden or lymph node metastases were more likely to experience improved survival with AC. Interaction plots indicated that AC was associated with a survival benefit beyond a TBS threshold of approximately six. Consequently, patients with either a TBS ≥ 6 or metastatic lymph nodes were classified as the AC “effective group” (*n* = 857, 60.7%), while the remaining patients were categorized as the “noneffective group” (*n* = 555, 39.3%). Notably, among the “effective group,” AC was associated with an improvement in 5-year OS [30.7% (95% CI 26.2–36.0) versus 33.0% (95% CI 26.9–40.5), *p* = 0.018]. In contrast, within the “noneffective group,” 5-year OS did not differ between patients who did receive and patients who did not receive AC [55.1% (95% CI 48.9–62.1) versus 58.7% (95% CI 49.8–69.2), *p* = 0.900].

## Future

The latest clinical guidelines recommend AC for all patients with ICC following curative-intent resection.^5^ Refining patient selection for AC could help minimize unnecessary treatment-related harm while maximizing therapeutic benefit, ultimately leading to a more individualized approach to postoperative management. Findings in the current study suggested that TBS and lymph node status may serve as useful criteria to guide AC decisions among patients with ICC. Future studies should focus on externally validating this classification in independent cohorts and assess whether omitting AC in the “noneffective group” is oncologically appropriate and does not compromise long-term outcomes. Additionally, in the “effective group,” further research is needed to validate the survival benefit of AC and determine whether the efficacy of AC remains consistent across different patient populations.
